# Influence of Powder Characteristics on Processability of AlSi12 Alloy Fabricated by Selective Laser Melting

**DOI:** 10.3390/ma11050742

**Published:** 2018-05-07

**Authors:** Rustam Baitimerov, Pavel Lykov, Dmitry Zherebtsov, Ludmila Radionova, Alexey Shultc, Konda Gokuldoss Prashanth

**Affiliations:** 1 “Micropowders Technologies” Laboratory, Research and Education Center “Aerospace Technologies”, South Ural State University (SUSU), Lenin Avenue 76, 454080 Chelyabinsk, Russia; baitimerovrm@susu.ru (R.B.); lykovpa@susu.ru (P.L.); shultcao@susu.ru (A.S.); 2 Research and Education Center “Nanotechnology”, South Ural State University (SUSU), Lenin Avenue 76, 454080 Chelyabinsk, Russia; zherebtcovda@susu.ru; 3 Faculty of Material Science and Metallurgical Technologies, Polytechnic Institute, South Ural State University (SUSU), Lenin Avenue 76, 454080 Chelyabinsk, Russia; radionovalv@susu.ru; 4 Department of Manufacturing and Civil Engineering, Norwegian University of Science and Technology, Teknologivegen 22, 2815 Gjøvik, Norway; 5 Erich Schmid Institute of Materials Science, Austrian Academy of Sciences, Jahnstraße 12, A-8700 Leoben, Austria; 6 Department of Mechanical and Industrial Engineering, Tallinn University of Technology, Ehitajate Tee 5, 19086 Tallinn, Estonia

**Keywords:** additive manufacturing, selective laser melting (SLM), AlSi12 alloy, powder, porosity

## Abstract

Selective laser melting (SLM) is one of the additive manufacturing technologies that allows for the production of parts with complex shapes from either powder feedstock or from wires. Aluminum alloys have a great potential for use in SLM especially in automotive and aerospace fields. This paper studies the influence of starting powder characteristics on the processability of SLM fabricated AlSi12 alloy. Three different batches of gas atomized powders from different manufacturers were processed by SLM. The powders differ in particle size and its distribution, morphology and chemical composition. Cubic specimens (10 mm × 10 mm × 10 mm) were fabricated by SLM from the three different powder batches using optimized process parameters. The fabrication conditions were kept similar for the three powder batches. The influence of powder characteristics on porosity and microstructure of the obtained specimens were studied in detail. The SLM samples produced from the three different powder batches do not show any significant variations in their structural aspects. However, the microstructural aspects differ and the amount of porosity in these three specimens vary significantly. It shows that both the flowability of the powder and the apparent density have an influential role on the processability of AlSi12 SLM samples.

## 1. Introduction

Selective laser melting (SLM) is one of the metal additive manufacturing (AM) techniques that allows the production of metallic parts from powder feedstock using layer-by-layer approach directly from a computer aided design model (CAD) [1,2,3]. In comparison with conventional manufacturing techniques (such as casting, forging, extrusion, and powder metallurgy), SLM has some undoubted advantages: possibility of fabricating nearly full density three-dimensional parts with complex shape [3,4,5,6]; high material utilization rates; minimal post-processing requirement; flexibility of fabricating complex shaped metal matrix composites; etc. [7,8,9,10]. However, the quality of SLM fabricated parts strongly depends on the laser processing parameters, building chamber atmosphere, powder bed preheating, and especially on the powder feedstock characteristics [11,12,13].

A significant amount of SLM research has been devoted to optimize the process parameters (laser power, scanning speed, scanning strategy, layer thickness, building chamber atmosphere, powder bed preheating temperature, and post processing heat treatment) and on numerical simulations [2,11,14,15,16]. On the other hand, only a few works have been devoted towards the influence of powder characteristics on the quality of SLM fabricated parts. Spierings et al. compared the SLM processing behavior of three 316L stainless steel powder batches with varying particle size distribution and found that fine particles are beneficial for high part densities, process productivity, and scan surface quality [17]. Similarly, Liu et al. has demonstrated that powder of 316L stainless steel with high content of fine particles result in higher powder bed density and in turn generates higher density parts under low laser energy intensity [18]. In addition, 316L powder with high volume of fine particles results in better surface quality [18]. Powder with a narrow particle size distribution leads to better flowability and hence packing density that generates parts with higher tensile strength and hardness. With respect to powder morphology, Li et al. found the gas atomized spherical 316L powder generates denser structure than water atomized irregular powder due to higher packing density and lower oxygen content [19]. Similar results were obtained also for AlSi10Mg alloy and IN738LC alloys [20,21,22]. Gu et al. had studied the SLM processing of three TiAl6V4 powder batches and found that one of the powder batches with significantly large amount of fine particles had resulted in significantly higher powder bed thermal conductivity in comparison with the other two powder batches [23]. Hence, different processing parameters may be required for differences in the powder morphology, powder distribution and possible chemical composition that may arise from different powder batches.

Researchers have also showed that the chemical composition of powder may sufficiently influence the SLM process and its process parameters. For example in Hastelloy X and IN738LC compositions, it has been found that the crack sensitivity during SLM process depends greatly on the amount of minor elements such as Mn and Si [22,24]. It has been shown in AlSi10Mg alloy that higher Si content has a positive impact on SLM processability, since Si has a positive influence on the absorptivity of laser energy by the powder bed [25]. Averyanova et al. compared the SLM processing of 17-4 PH martensitic stainless steel employing two different powder batches with minor variations in the chemical composition [26]. Further, it has been demonstrated that the minor variations in the chemical composition did not have a significant influence on the densification behavior but it severely influences the microstructure and in turn the mechanical properties [26]. In general, all the above-mentioned works demonstrate that the powder characteristics are critical for the SLM processability (process parameters) and has a significant influence on the quality of the SLM parts. The powder characteristics may change between suppliers and even from batch to batch within the same supplier. Further investigation on the influence of the powder characteristics on the SLM processability is necessary in order to use SLM successfully as a reliable alternative to conventional metal manufacturing techniques.

Al–Si and Al–Si–Mg alloys are widely used in automotive and aerospace industries due to their low density, good mechanical properties, high wear, and corrosion resistance [27,28,29]. The majority of the SLM research on Al-based alloys are devoted towards the processing of Al–Si and Al–Si–Mg alloys [30,31,32]. However, Al–12Si alloy has attracted increased attention recently, because, AlSi12 is a near eutectic alloy, that is characterized by excellent castability and good specific strength [33,34]. The effect of heat treatment on the SLM-processed AlSi12 specimens were studied extensively and it has been found that heat treatment significantly alters the microstructure and hence affects the mechanical properties [35,36,37,38,39]. In addition, it has been reported that the protective gas used during the SLM process does not affect the densification behavior of AlSi12 significantly. However, they marginally influence the ductility [33]. The tribological and corrosion behavior of AlSi12 specimens fabricated by SLM (as-fabricated and heat-treated) were compared with the cast alloy and the influence of cooling rate on the microstructure and the properties has been established [40]. Since Al-12Si processed by SLM is well established and it is an easy system to be processed by SLM without the formation of complex intermetallics and with good fluidity, we use this (Al-12Si) system as a starting material for our investigation. In this paper, we study the influence of three different AlSi12 powders (with varying particle size distribution, morphology, and chemical composition) on the processability by SLM.

## 2. Materials and Methods

### 2.1. SLM System

**Figure 1:**
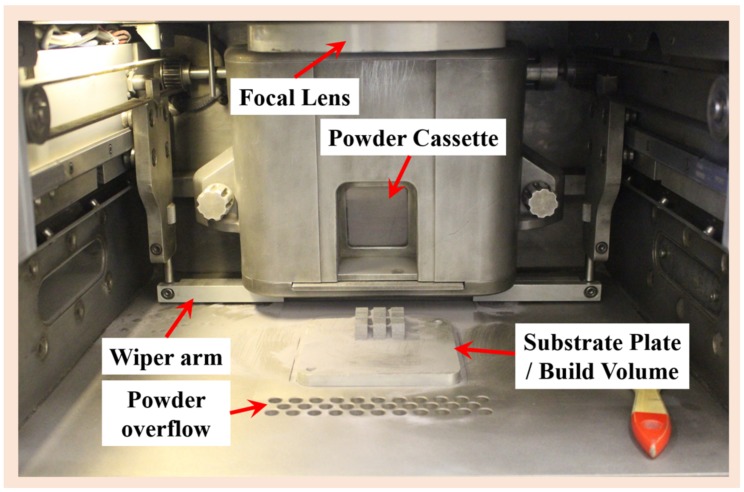
An image of the Sinterstation^®^ Pro DM125 selective laser melting (SLM) system showing the building chamber and its parts including substrate plate.

The SLM experiments were carried out on a Sinterstation^®^ Pro DM125 SLM System from 3D Systems, Inc. (Rock Hill, SC, USA) (shown on Figure 1). Sinterstation^®^ Pro DM125 SLM System is equipped with a fiber laser (max. power 200 W) (Southampton, UK) with a laser beam diameter of 35 μm at the powder surface. This also includes an automatic powder spreading system, an inert gas (Ar) protection system and a powder bed preheating system. The layer thickness can be varied between 20–100 μm and the maximum laser scan speed of 1000 μm/s can be achieved.

### 2.2. Materials

Three different batches of AlSi12 alloy powder from three different suppliers were taken as the raw materials for this work. The batches were designated with letters A, B and C. Powders A and B were gas atomized using argon whereas powder C were gas atomized using air. Powder A was specially produced for SLM Solution from MTT Technologies Ltd. (Stone, UK). Powder B was produced by Ashinskiy Metzavod PAO (Asha, Russia) (“Metal powders”) and Powder C was produced homemade by authors using URM-001 powder atomizer in South-Ural State University (Chelyabinsk, Russia) [41]. A detailed information about the production of powder C can be found elsewhere [42]. Each powder batch was characterized in terms of particle size distribution, morphology, chemical composition, flowability and apparent density. The particle size distributions were measured using an Occhio 500 nano particle analyzer (Occhio SA, Liège, Belgium). The morphology and chemical composition of the powder was examined using a JEOL JSM-7001F scanning electron microscope (SEM) (JEOL Ltd., Tokyo, Japan) equipped with an Oxford INCAX-max 80 energy dispersive spectrometer (EDS). The chemical composition was determined using ONH-2000 analyzer (ELTRA GmbH, Haan, Germany). The flowability was measured using the Hall flowmeter funnel according to the ASTM standard [43]. The apparent density was measured using standard ASTM procedures [44,45].

### 2.3. SLM Process Parameters

A density optimization process was carried out using Powder A, in order to determine the optimal process parameter for Al-12Si samples using the SLM process. Hence, nine 10 × 10 × 10 mm^3^ specimens were fabricated by varying point distance (PD), hatch spacing (HS) and exposure time (ET), whereas the laser power (P) was fixed at the maximum value (200 W). This is because aluminum alloys have low absorptivity for laser radiation [46]. Hence, maximum power is required to have effective melting and to increase the speed of the fabrication process. Layer thickness (LT) was fixed at 50 μm, considering the powder particle size distribution. Stripe hatch scanning strategy was used to fabricate the SLM samples with a stripe width of 4 mm and 37° stripe angle increment. The combination of processes parameters observed for the specimens with the lowest porosity was used for further studies with Powder B and Powder C. Each powder batch was dried at 100 °C for 60 min to remove the possible moisture skin from powder particle surface and this procedure was carried out before each SLM process [47]. Three 10 mm × 10 mm × 10 mm cubic specimens were fabricated from each powder batch and the processing conditions were maintained to be the same. 5 mm support structures were built between the specimens and aluminum substrate plate to facilitate easy removal of the specimens. Argon atmosphere with slight overpressure conditions inside the building chamber was used to avoid oxygen contamination during the SLM process. Oxygen content inside the building chamber was maintained below 500 ppm.

### 2.4. Porosity and Microstructure Testing

After the fabrication of parts by SLM, SEM was used to examine the top surface morphology of the specimens. Then the specimens were cut both parallel and perpendicular to the building direction, subsequently mounted, grinded and polished following the standard metallographic procedures. Non-etched cross-sections were examined by Olympus GX51 (Olympus Corporation, Tokyo, Japan) inverted optical microscope (OM) at 20× magnification for porosity determination and according to the procedure described by Spierings et al. [48]. Porosity was calculated by area fraction analysis from the OM micrographs using GIMP 2.8.22 software (version: 2.8.22). The porosity values reported are average values of the three specimens from each powder batch using a 95% confidence interval. From the porosity calculations, the relative densities were calculated simultaneously. The polished cross-sections were then etched using Keller’s reagent (95 mL water, 2.5 mL HNO_3_, 1.5 mL HCl, 1.0 mL HF) and the microstructural examination was carried out using OM and SEM.

## 3. Results and Discussion

### 3.1. Powder Characterization

**Figure 2:**
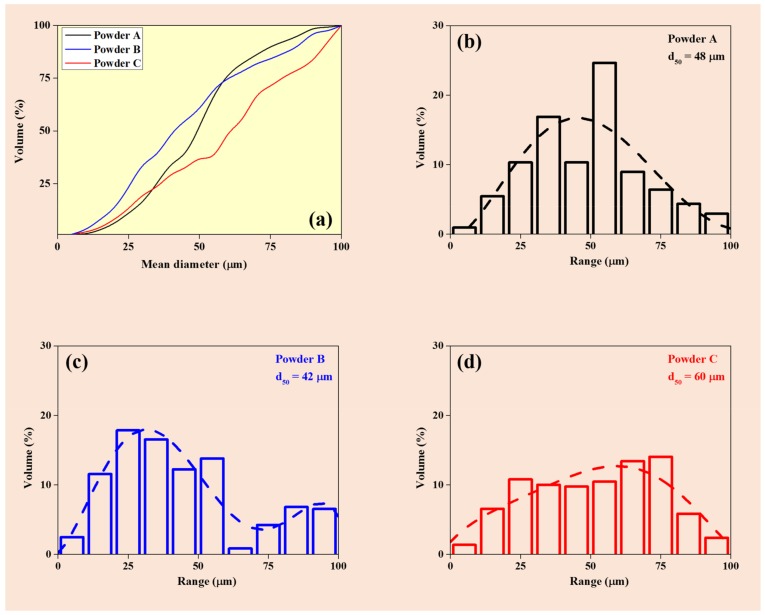
Particle size distribution for the powders (**a**) comparison of powders A, B and C as a function of mean diameter and Vol. % and particles size distribution bar charts for (**b**) powder A, (**c**) powder B and (**d**) powder C.

The chemical composition of the three different AlSi12 powder batches evaluated in this study are presented in Table 1. The powder batches B and C have almost the same Si content (13.13 and 13.16 wt %, respectively) in contrast to the powder A, where the Si content is marginally less around 12.48 wt %. Powder C has increased amounts of Fe and Cu, which may be the residuals from the previous atomization process. As expected, air atomized powder C contents have more oxygen than the other powder batches (A and B). Hence, the amount of Al in powder batch C is slightly reduced than the other two batches. All three powder batches have significantly low amounts of N content (<0.0016 wt %). The particle size distributions of each AlSi12 powder batch evaluated in this study are presented in Figure 2. It has been observed from Figure 2 powder B has large amount of fine particles (<25 μm) than powder batches A and C (where they have similar volume fractions below 25 μm). However, with increasing the powder size beyond 50 μm, powder A and B has similar volume fractions, which is much higher than powder C. This suggests that both powder A and B have relatively fine powder particles than powder C. The d_10_, d_50_, d_90_, particle size distribution span, apparent density, and flowability values are summarized in Table 2. The span value was calculated using the following equation [22]:

(1) $$\mathrm{span}=\frac{\left(d_{90}-d_{10}\right)}{d_{50}}.$$

Engeli et al. [22] has demonstrated that the span of the distribution has a significant influence on the span of the flowability. Even in the present study, it can be clearly observed that the span of the powder distribution has a significant influence on the flowability. The lower the span of the powder distribution, the better the flowability of the powder. When the span of the powder distribution increases, the flowability of the powder gets hampered. It can be observed in Table 2 that the particle size distribution, d_10_, d_50_, and d_90_ values are very different for the three powder batches. The effective layer thickness (*LT_eff_*) is real layer thickness that is bigger than the nominal layer thickness. Such differences in the layer thickness are observed due to shrinkage of the powder layer during the laser melting process [49]. The theoretical *LT_eff_* values for each powder bed was calculated as [50]:

(2) $$LT_{eff}=LT\cdot \;\frac{\rho_{metal}}{\rho_{powder}}$$

where $\rho_{powder}$ and $\rho_{metal}$ are the density of the powder layer and the fabricated solid metal, respectively. Theoretical density of AlSi12 alloy was accepted as 2.66 g/cc [51] and the apparent density was denoted as $\rho_{powder}$. It can be observed from Table 2 that the *LT_eff_* increases with the d_50_ values and also depends greatly on the apparent density (or the packing density of the powder bed). The wider the particle size distribution or coarser the powder particles, the more apparent density decreases and hence the *LT_eff_*. The only other parameter that shows an opposite trend for the increase the powder particle size and the size distribution is the flowability.

### 3.2. SLM Parameter Optimization

**Figure 3:**
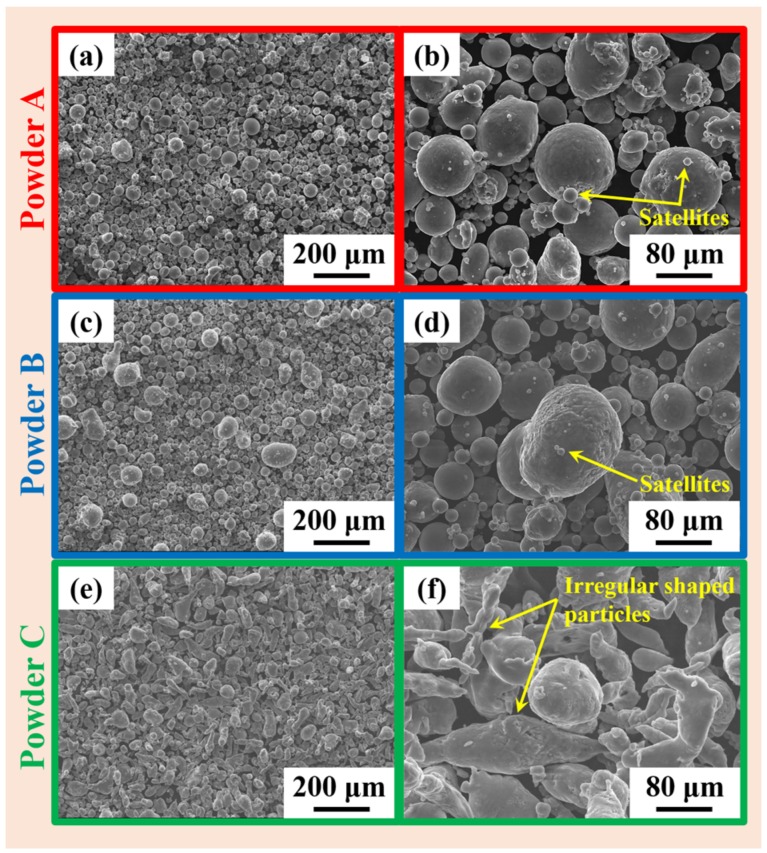
Scanning electron microscope (SEM) micrographs of the three different AlSi12 powders produced from three different powder batches (**a**,**b**) powder A, (**c**,**d**) powder B and (**e**,**f**) powder C, showing the particle morphology.

Powder B has the lowest d_10_ and d_50_ values, suggesting that it contains finer powder particles than the other two batches. Hence, powder B shows better apparent density. On the other hand, the flowability of the powder is significantly hampered. Generally, finer powder particles have a tendency to form agglomerates that reduces the flowability of the powder. However, at the same time, fine particles fill the void between two coarse particles and thereby help in increasing the powder layer density [52]. Powders A and C exhibit similar d_10_ values but the d_50_ value of powder C is much higher than powder A. This suggest that powder C has relatively coarser particles than powder A and is expected to have good flowability among the three powder batches. On the other hand, powder A shows better flowability than powder C, d_50_ of powder A is smaller than powder C. This effect can be attributed to the good spherical nature of the powder particles in batch A as compared to powder batch C (as shown in Figure 3). It may be observed from Figure 3 that the powder particles get less spherical as we move from powder A to powder C, which may be attributed to the characteristics of the initial production process and the nature of the inert gas used during powder manufacturing. Hence, the best flowability was found for the powder A, the powder with almost spherical nature among the lot. The morphology of the powders not only affect the flowability but also the apparent density. From Figure 3, it can be seen that powder C has more irregular shaped powder particles, which hampers the packing of the powders and hence, leads to the least apparent density among the three powders batches. The irregular shape of the powder C is attributed to the oxidation of the surface during atomization process (in air jet) [53,54,55,56,57,58]. Powder B has the spherical morphology with a certain amount of satellites on the surface of large particles like powder A, which is typical for gas-atomized powders [59].

**Figure 4:**
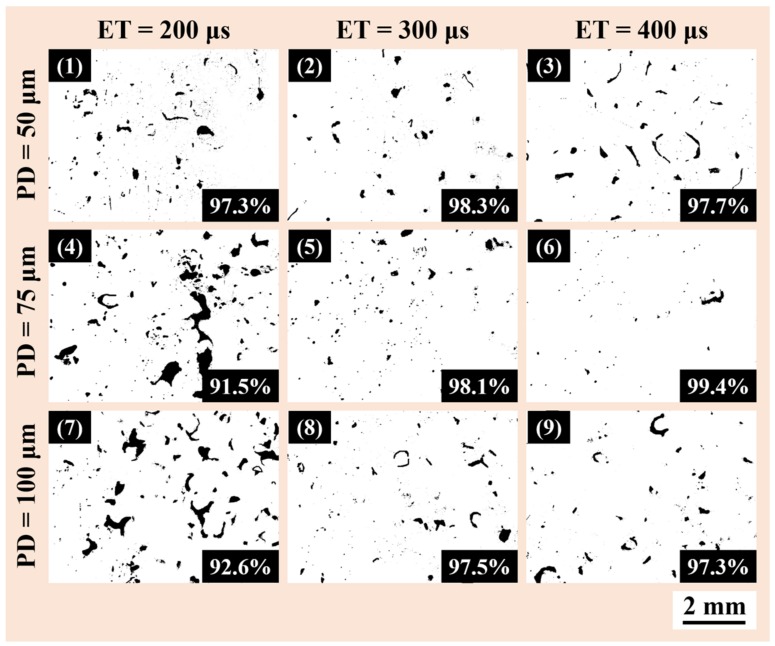
Porosity evolution in AlSi12 specimens fabricated from powder batch A, processed using different combination of point distance (PD) and exposure time (ET). The relative density value for each specimen is indicated in the left bottom corner of the corresponding picture.

The porosity evolution in AlSi12 specimens fabricated from powder A, processed using different combinations of point distance and exposure time are shown in Figure 4. It has already been demonstrated that laser power has the most significant influence among the various process parameters available and hence, we have kept it as a maximum [11]. Another reason for keeping the laser power maximum is to have a relatively faster production rate. With 200 μs as the exposure time (ET), large uniformly dispersed keyhole pores were observed though out the sample surface. The keyhole pores are generally formed due to lack of sufficient energy available to melt the powder bed. Hence, the melt pool cannot flow as required leaving to the formation of irregular shaped keyhole pores. With increasing the ET, the amount of keyhole pores decreases significantly. On the other hand, it has been observed that with decreasing point distance (PD) the amount of porosity in the AlSi12 samples decreases. A best relative density of 99.4% is observed for a sample with the maximum ET (400 μs) and moderate PD (75 μm). This best parameter combination has been used to fabricate samples from powder batches B and C (as shown in Table 3).

### 3.3. Influence of Powder Properties on SLM Processibility

**Figure 5:**
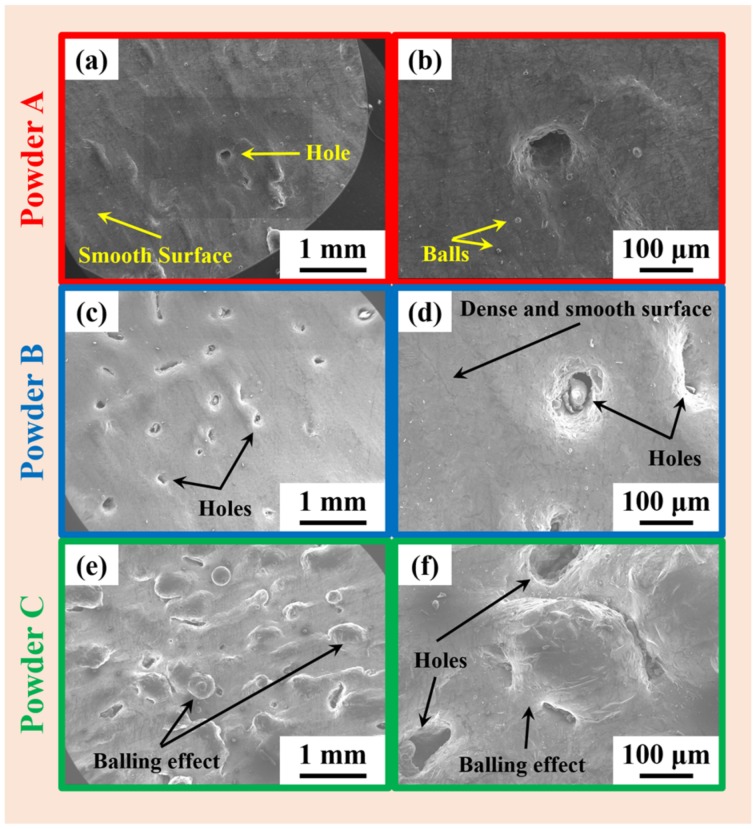
SEM images of top surface morphology of SLM fabricated specimens from the three different powder batches (**a**,**b**) powder A, (**c**,**d**) powder B and (**e**,**f**) powder C.

AlSi12 samples fabricated from all the three different powder batches processed under equal conditions have shown different behavior during SLM. Figure 5 shows the SEM microstructures of the top surface morphology of the AlSi12 SLM samples fabricated from different powder batches. The top surface of the specimen fabricated from the powder A show a dense and smooth morphology almost without inclusions, except for the tiny metallic balls that form from the splashing of small liquid droplets arising from the fast moving laser beam [20]. In addition, one or two (countable) number of holes were also observed. On the contrast, the samples prepared from powder B show a large amount of surface imperfections in the form of holes with dense and smooth areas between the holes. Powder B has a poor flowability due to large volume of fine particles, hence there is a difficulty in the formation of a homogenous/uniform and dense powder layer [49]. This results in a powder bed with non-uniform distribution of powder and hence the holes are formed where there is a scarcity of powder during melting. The surface of the SLM AlSi12 samples prepared from powder C show the presence of both holes and large spherical objects (>100 μm in diameter). The large spherical particles are the result of balling effect [53]. “Balling” occurs due to instability in the melt pool. This instability in the melt pool is due to the high surface tension gradients resulting from Marangoni convention effect [54,60]. Powder C has the highest LT_eff_ value (among the three powder batches) and hence the low apparent density. Accordingly, low-density powder beds have low effective thermal conductivity [55], because of the presence of voids (air pockets) in the powder bed. Hence during laser melting, the melt pool will be shallow at some places than other, and the melt pool is over heated in these places. This overheating of the powder bed/melt pool at isolated places result in high temperature gradients and hence results in “balling” effect. The relative densities of the AlSi12 SLM specimens fabricated from the different powder batches are presented in Table 4. As expected, the specimens from the powder A have the highest relative density compared to powders B and C, corroborating with the SEM images in Figure 5, which again boils down to the spherical nature of the powder, flowability, and apparent density, which are closely connected to each other.

**Figure 6:**
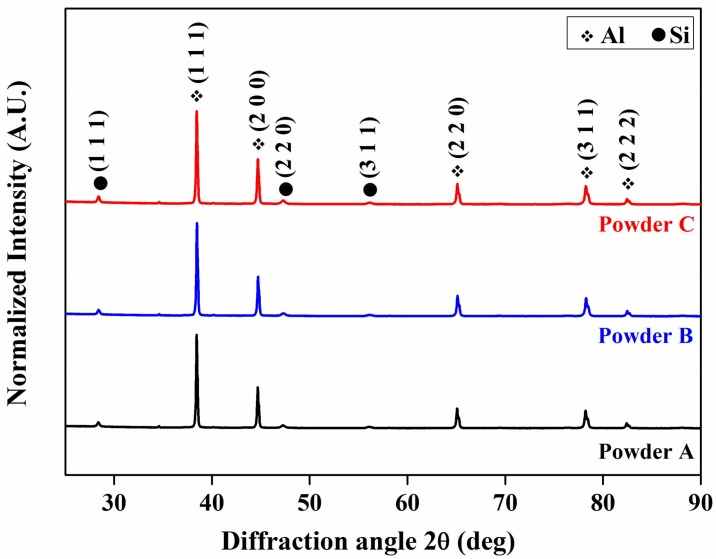
X-ray diffraction pattern of the Al-12Si samples produced from three different powder batches.

Figure 6 show the XRD patterns of the AlSi12 SLM samples prepared from different powder batches. As expected the XRD patterns show the crystalline peaks of two distinct phases: Al and Si. The intensity of the Si peaks are drastically reduced, even though the solubility of Si is limited in Al at room temperature. The reduced intensity in the Si peaks are due to the formation of the supersaturated solid solution of Si in Al, due to high cooling rates observed during the SLM process [28,35]. This is very similar to the other published reports, where the supersaturation of Si in Al is well established [28,31,35,36]. On the other hand, there is no crystallographic texture observed in these samples as compared with published reports [28,31,35]. The absence of texture in these samples can be attributed to the laser scan strategy used during the fabrication process, where the scan rotation of 37° is used between the layers unlike the published reports, where a scan rotation of ~73° is used [28,35,61]. However, it is interesting to note that the AlSi12 SLM samples prepared from the three different powder batches show similar XRD patterns with no significant difference between them. It suggests that the changes in the minor composition, morphology of the AlSi12 particles and the production process do not have a significant influence on the structural properties of these SLM fabricated AlSi12 samples.

### 3.4. Influence of Powder Properties on Microstructure of SLM Fabricated Specimens

**Figure 7:**
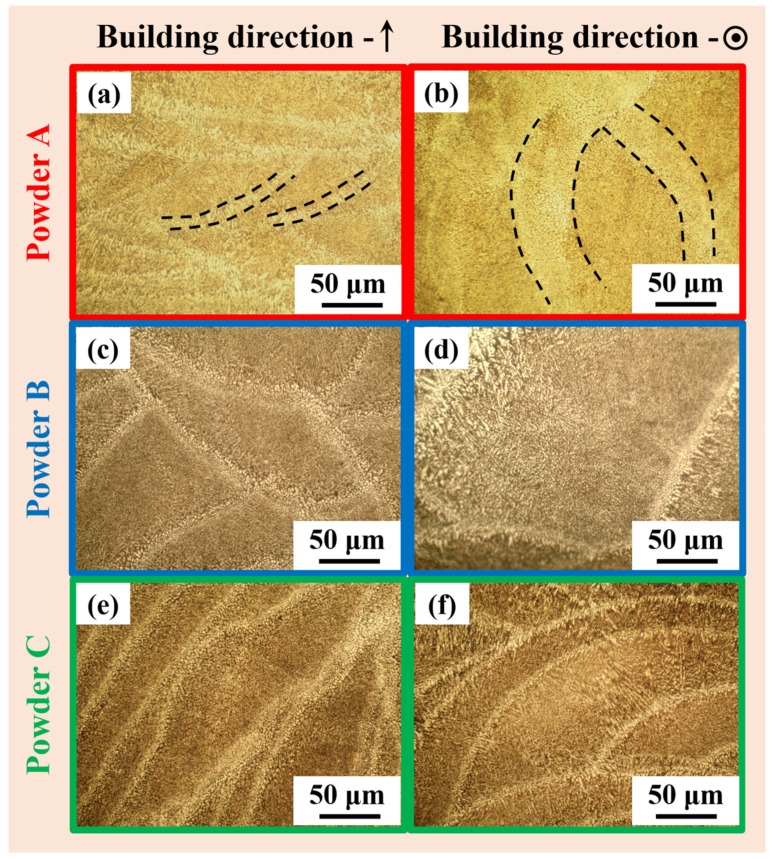
Optical microscopy images of the AlSi12 specimens fabricated by SLM from the three different powder batches (**a**,**b**) powder A, (**c**,**d**) powder B and (**e**,**f**) powder C, showing hatches and hatch overlaps.

**Figure 8:**
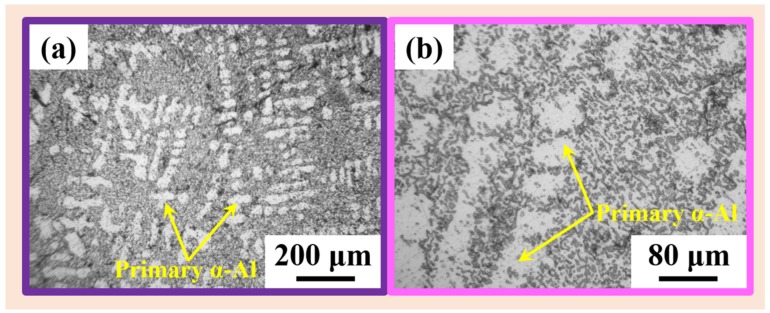
Optical microstructure of cast AlSi12 specimen at (**a**) lower magnification and (**b**) higher magnification, showing the presence of primary α-Al and Al-Si eutectic microstructure.

Figure 7 presents the OM micrographs describing the microstructure of the SLM fabricated AlSi12 specimens from the three different powder batches. It can be observed from these microstructures that all the AlSi12 samples exhibit features corresponding to SLM layer-by-laser melting strategy, which is significantly different from the AlSi12 cast microstructures both in terms of length scales and morphology and distribution of the phases. In order to compare the SLM microstructure with its cast counterpart, the microstructure of the AlSi12 cast sample is shown in Figure 8. The cast microstructure shows a hypoeutectic morphology with primary α-Al dispersed in continuous Al–Si eutectic structure [35], which is completely different from the layered microstructure observed for the SLM samples (Figure 7). In addition, the AlSi12 SLM samples show the presence of inhomogeneity in the microstructure, where coarse features are observed along the hatch overlaps and finer morphology along the core of the hatches [35,62]. Nevertheless, no significant variation in the features were observed between the three samples fabricated from three different powder batches, especially at this length scale.

**Figure 9:**
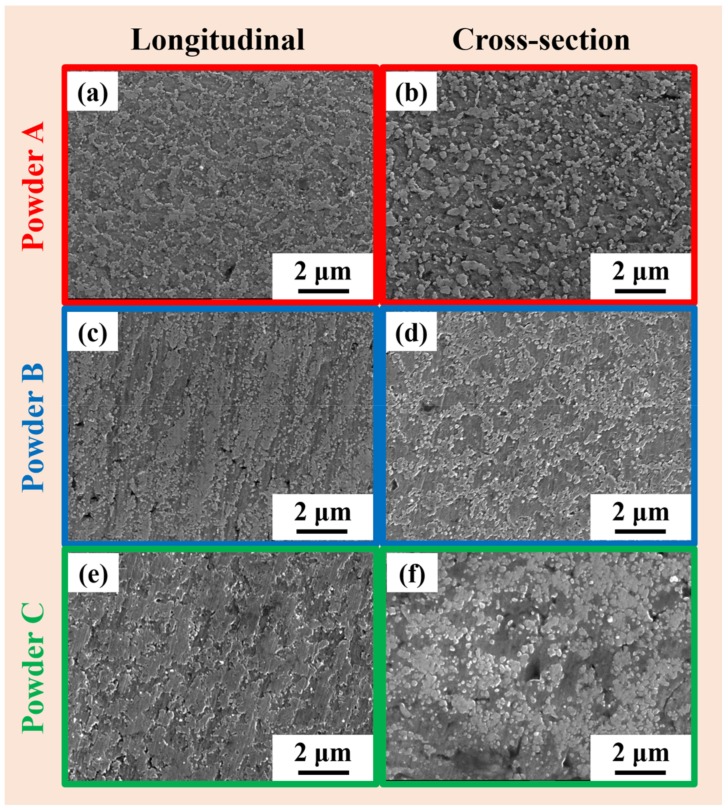
Microstructure of AlSi12 specimens fabricated by SLM from the different powder batches (SEM) (**a**,**b**) powder A, (**c**,**d**) powder B and (**e**,**f**) powder C.

Figure 9 shows the high magnification SEM images of the AlSi12 SLM specimens produced from the three different powder batches. It can be observed from Figure 9 that the specimens exhibit a sub-micron cellular morphology, which is typical for AlSi12 SLM samples, where the core of the cells are rich in Al and the cell boundaries are rich in Si [35]. In contrast with specimen A, which has very homogeneously dispersed Si rich grains, the specimens B and C have micron-sized Si rich areas. Probably, that can be explained by the higher content of Si in the powders B and C. However, distinctive variation in the microstructures can be observed between specimens prepared from powder A and from powder batches B and C. In specimens prepared from powder A, the Si phase is more homogeneously distributed and on the other hand, the specimens fabricated from powders B and C have some micro-sized Si rich areas. Such micro-size Si rich areas may be a resultant of the presence of a slightly higher Si concentration (slightly more than the eutectic composition), which can be noted from Table 1. Since, the Si concentration in the samples produced from powder B and C, crosses the eutectic mark of 12.6 wt %, the excess Si is precipitated in the form of sub-micron particles though out the samples, but embedded on a near cellular microstructure. Hence, these results suggest that variations in the powder batch (in terms of powder morphology, powder size, powder size distribution, and chemical composition), will have a significant influence in the microstructural features including porosity levels in the SLM prepared samples. The porosity levels in these samples can be modified by having minor variation in the process parameter, essentially suggesting that process parameter optimization has to be carried out, whenever there is a change in the powder batch.

## 4. Conclusions

The influence of the starting powder characteristics on SLM processability of AlSi12 alloy was studied in this paper. The main conclusions are summarized as follows:

(1) High density AlSi12 SLM samples were fabricated using the following optimized process parameters: 200 W laser powder; 50 μm layer thickness; 75 μm point density; 75 µm hatch spacing; and 400 μs exposure time using fiber laser with a 35 μm laser beam diameter.
(2) Both the flowability and powder layer density have an important influence on the SLM processability of AlSi12 powder. The powder batch with good flowability and apparent density combination have shown the best SLM processability.
(3) The powder batch with near spherical morphology of particles had slightly reduced flowability than the powder with near spherical particles and hence low apparent density. As a result, the SLM process leads to the famous “balling” effect and high porosities observed in the samples.
(4) The powder batch with reasonable spherical morphology of particles and with high volume of finer particles exhibited a very bad flowability, which is attributed to the formation of agglomerates. Using such a powder leads to high porosity levels in the SLM fabricated AlSi12 samples.

## Figures and Tables

**Table 1:** Chemical composition of the standard AlSi12 alloy powders from different batches produced from different process routes.

Powder Batch	Elements (wt %)				Gas Content (wt %)	
	Al	Si	Fe	Cu	O	N
A	86.63	12.48	0.64	0.24	0.0796	0.0010
B	86.28	13.13	0.47	0.05	0.0659	0.0008
C	85.35	13.16	0.93	0.46	0.0963	0.0016

**Table 2:** Characteristic values for the particle size distribution, the flowability, the apparent density and the theoretical effective layer thickness for the different powder batches A, B and C.

Powder Batch	d_10_ (μm)	d_50_ (μm)	d_90_ (μm)	Span (-)	Apparent Density (g/cc)/(%)	Flowability (s/50 g)	*LT_eff_* (μm)
A	24	48	71	0.98	1.20/45.1	65.4 ± 0.41	111
B	17	42	85	1.62	1.28/48.1	No flow	104
C	22	60	94	1.20	1.05/39.5	72.2 ± 0.63	127

**Table 3:** SLM processing parameters.

Power (W)	Layer Thickness (μm)	Point Distance (μm)	Exposure Time (μs)	Hatch Space (μm)
200	50	75	400	75

**Table 4:** Relative density of SLM specimens fabricated from the three different powder batches.

**Relative Density (%)**	**Powder A**	**Powder B**	**Powder C**
	99.4 ± 0.3	95.6 ± 1.6	94.4 ± 2.3
